# Supplementary material for: Cinnamon Oil Inhibits Penicillium expansum Growth by Disturbing the Carbohydrate Metabolic Process
Source: J Fungi (Basel). 2021 Feb 9;7(2):123. doi: 10.3390/jof7020123 (PMC7915993; doi:10.3390/jof7020123)
Supplement: Supplementary file 1 [file jof-07-00123-s001.zip › 6.jof-1092000-sent proof-supplementary/6.jof-1092000-sent proof-supplementary.pdf]

Supporting Information

Table S1 Information of the differentially expressed proteins in *P. expansum* spores under 0.25 mg L^{-1} cinnamon oil stress after 6 h of culturing by iTRAQ analysis.

Accession: the accession number of non-redundant protein database of NCBI; Expt. Mr: Experimental molecular mass; Coverage: Amino acid sequence coverage for the identified proteins; Sequence: The sequence information of identified peptides; Score: Mascot scores are statistically significant ($p<0.05$); Control/Cinnamon oil: Fold change of relative abundance of DEGs at control group versus cinnamon oil treatment group; COG: Clusters of Orthologous Groups of proteins; KEGG: The Kyoto Encyclopedia of Genes and Genomes; EC: Enzyme Commission; GO: Gene Ontology.

Table S2 Primer pairs used for qRT-PCR analysis

Name	Number	NCBI accession	Description	Sequence (5'-3)	Product length (bp)
PeCO1	DEP867	gi|700451849|gb|KGO41868.1|	Aldo/keto reductase	Forward: GGTTGCCTCCCAAGGAATGA<br>Reverse: AACCATAGCCTGATGCACGG	178
PeCO2	DEP861	gi|700486419|gb|KGO72046.1	Glucose/ribitol dehydrogenase	Forward: GAGGGACTTTGCTCGTGTCA<br>Reverse: GGGTCACTTTGACGACCCAT	126
PeCO3	DEP853	gi|700477337|gb|KGO64934.1|	Polyketide synthase, enoylreductase	Forward: TATCCAGGGTTCGCTCGTTG<br>Reverse: AGTCTCGACGCCATCTTTGG	121
PeCO4	DEP847	gi|700488368|gb|KGO73655.1|	Short-chain dehydrogenase/reductase SDR	Forward: CGGACGGACGTTGGTAGAAA<br>Reverse: GCCGAGTTGCTGCTTTCAAT	159
PeCO5	DEP827	gi|700475873|gb|KGO63722.1|	Pyruvate carboxyltransferase	Forward: ACCACCACAAGCGCAATCTA<br>Reverse: CCTCCGTAGTTGTCGCTACC	146
PeCO6	DEP818	gi|700477787|gb|KGO65288.1|	Acetate/Proprionate kinase	Forward: GACGCGTCGATCTCTGGAAT<br>Reverse: ACAGCGCTGCAAGAGGTATT	135
PeCO7	DEP811	gi|700485292|gb|KGO71124.1|	Bifunctional 6-phosphofructo-2-kinase/fructose-2, 6-bisphosphate 2-phosphatase	Forward: CGCAAAGCAATCGTTGACCA<br>Reverse: GGGTCCTGGCCTTTGTAGTC	146
PeCO8	DEP794	gi|700460677|gb|KGO49984.1|	Fructose-1,6-bisphosphatase class 1/Sedoheputulose-1,7-bisphosphatase	Forward: GGACTCGCTGGTTCCTCAAA<br>Reverse: AGACAACAGCGTAACGAGCA	178
PeCO9	DEP789	gi|700470445|gb|KGO58790.1|	Histidine phosphatase superfamily, clade-1	Forward: GAGGTTACGGAGGCGGTTAG<br>Reverse: TCTCCACCGGGACATCCATC	134
PeCO10	DEP784	gi|700469756|gb|KGO58135.1|	Succinate CoA transferase	Forward: CTCTTCGACAGCGGAAACCT<br>Reverse: ATGATCTCGGGGGAGTTGGA	149

PeCO11	DEP781	gi|700477299|gb|KGO64915.1|	Aldehyde dehydrogenase, N-terminal	Forward: CCGGGTACTTCATCCATCCG<br>Reverse: AGGACATCGTCCTCGTCAGA	121
PeCO12	DEP776	gi|700471576|gb|KGO59833.1|	Pyridoxal phosphate-dependent transferase, major region, subdomain	Forward: ACCGACACTCGCTTTGTGAT<br>Reverse: ACTAGCCGCATCAACGTGAA	180
PeCO13	DEP764	gi|700478031|gb|KGO65485.1|	Glucose-6-phosphate dehydrogenase, NAD-binding	Forward: AGCTTGTCATCCGTGTCCAG<br>Reverse: TCACGGACGAAGTTGGAGTG	199
PeCO14	DEP752	gi|700472832|gb|KGO60973.1|	Glycoside hydrolase, superfamily	Forward: GCCAAAGACCACGTTGTGAC<br>Reverse: GATCCAGTGGACTTGCCCTC	170
PeCO15	-	XM_016739499	CreA, Zinc finger, C2H2	Forward: AGAACATGACCCGTTCGCAT<br>Reverse: TGGGTGAGTTGGGACGAGAG	81

11

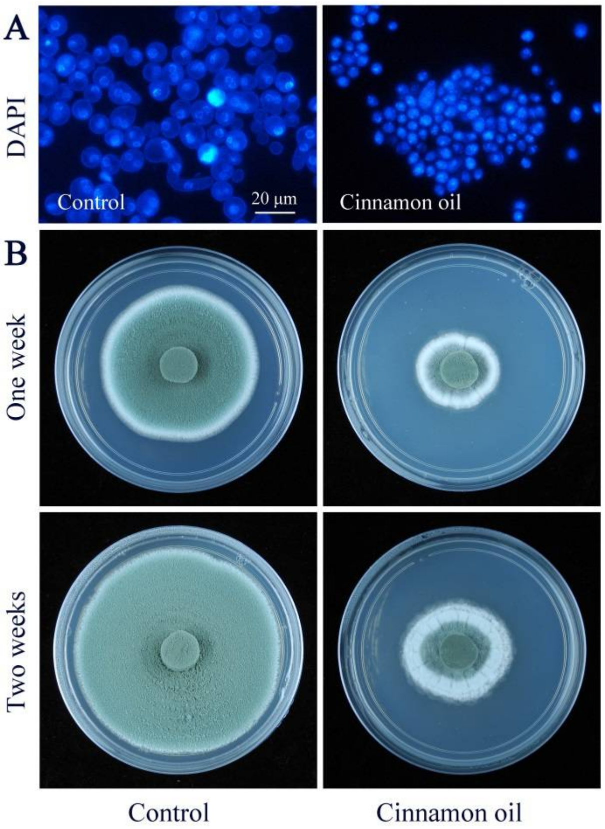

Figure S1. (A) Microscopic observation of *P. expansum spores* with DAPI staining after 6 h of culturing under 0 or 0.25 mg L^{-1} cinnamon oil stress. (B) Effects of 0.25 mg L^{-1} cinnamon oil on colonial morphology of *P. expansum*.

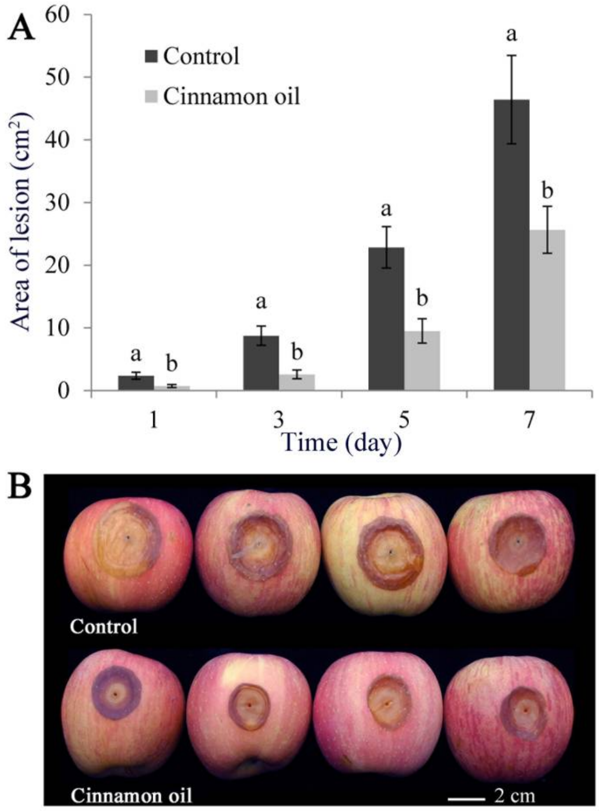

Figure S2. (A) Effects of 0.25 mg L^{-1} cinnamon oil on blue mold rot caused by *P. expansum* in apple. Bar represents the standard deviation of the means of three independent experiments. Low case letters indicate significant differences at $p<0.05$ at each time point. (B) The apples inoculated by *P. expansum* spores with 0 or 0.25 mg L^{-1} cinnamon oil after 7 days of storage at 25 ^{o}C.

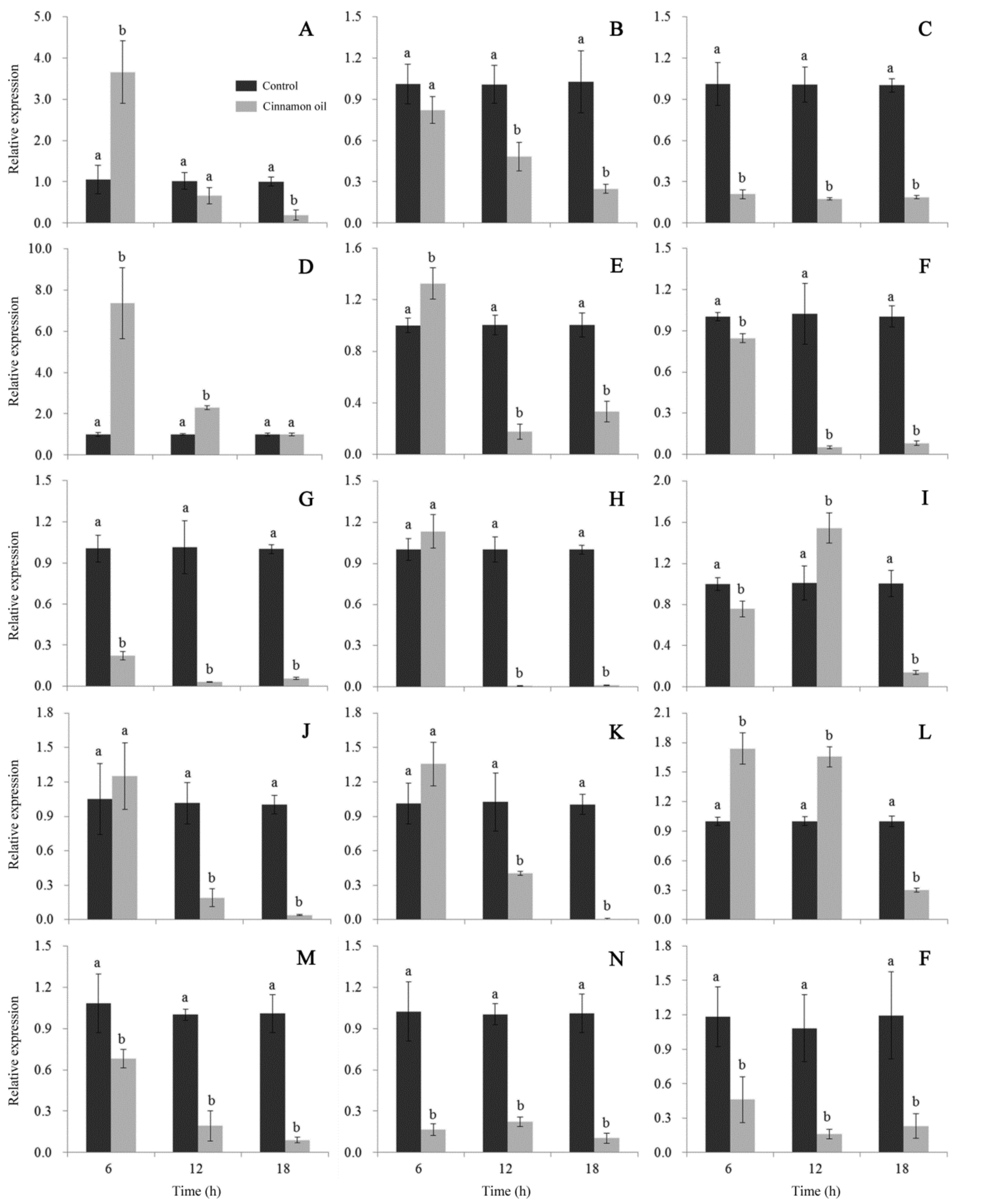

Fig. S3. Effects of 0.25 mg L^{-1} cinnamon oil on the expression of genes involved in patulin biosynthesis in *P. expansum*. Relative expression levels of PatA to PatO (A to O) are determined by qRT-PCR and the *β-tubulin* is used as the internal control. Bar represents the standard deviation of the means of three independent experiments. Low case letters indicate significant differences at *p*<0.05 at each time point.

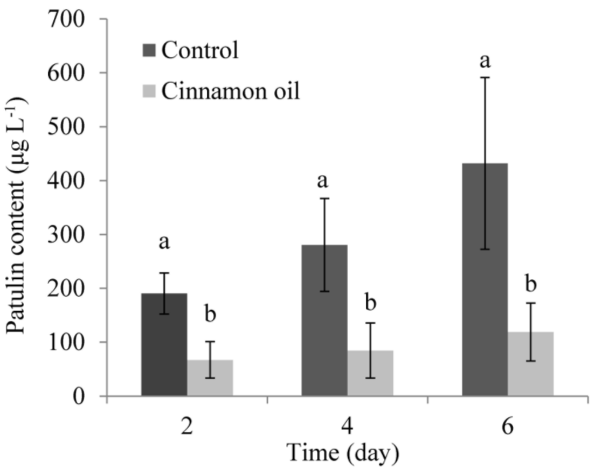

Figure S4. (A) Effects of 0.25 mg L^{-1} cinnamon oil on patulin production of *P. expansum.* Bar represents the standard deviation of the means of three independent experiments. Low case letters indicate significant differences at $p<0.05$ at each time point.

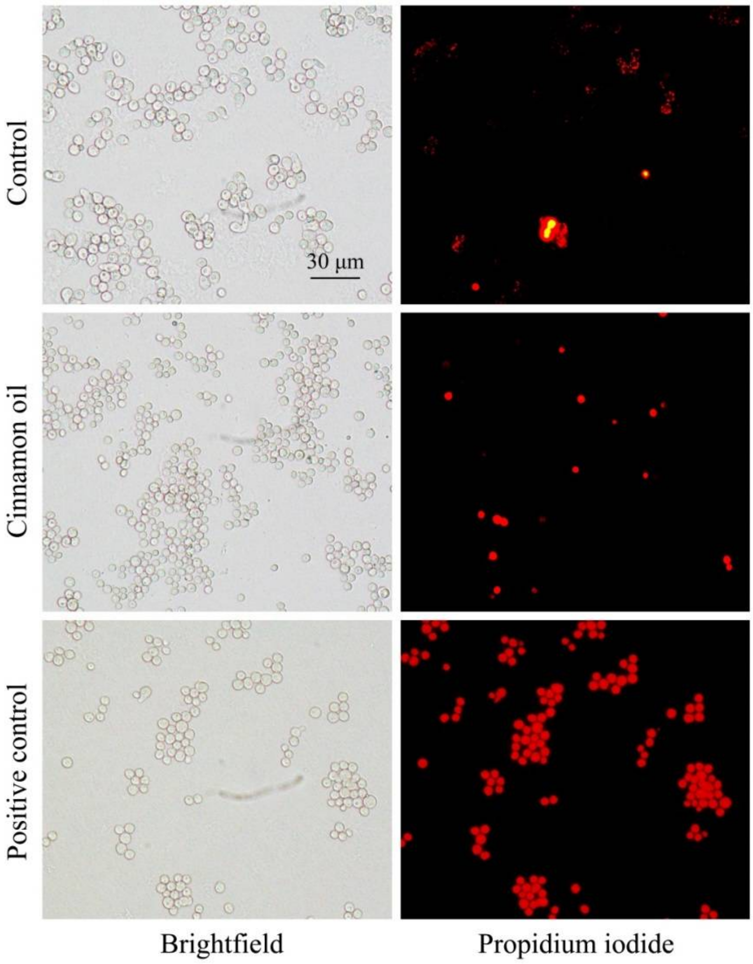

Figure S5. Effect of 0.25 mg L^{-1} cinnamon oil on the membrane integrity of *P. expansum* spores. Spores were cultured at 25 °C in PDB medium supplemented with 0 (negative control), 0.25 mg L^{-1} cinnamon oil. After 6 h of culture, half of the cinnamon oil-treated spores were incubated in boiling water for ten minutes (as positive control). Then, all the spores were stained with the prodium iodide and observed with a fluorescence microscope.
